# Retrieval, Monitoring, and Control Processes: A 7 Tesla fMRI Approach to Memory Accuracy

**DOI:** 10.3389/fnbeh.2013.00024

**Published:** 2013-04-08

**Authors:** Uda-Mareke Risius, Angelica Staniloiu, Martina Piefke, Stefan Maderwald, Frank P. Schulte, Matthias Brand, Hans J. Markowitsch

**Affiliations:** ^1^Physiological Psychology, University of BielefeldBielefeld, Germany; ^2^Neurobiology and Behavioral Genetics, University Witten-HerdeckeWitten, Germany; ^3^Erwin L. Hahn Institute for Magnetic Resonance ImagingEssen, Germany; ^4^General Psychology: Cognition, University of Duisburg-EssenDuisburg, Germany; ^5^Institute for Advanced ScienceDelmenhorst, Germany; ^6^Center of Excellence Cognitive Interaction Technology, University of BielefeldBielefeld, Germany

**Keywords:** memory confidence, memory retrieval, monitoring, movie, real-life events

## Abstract

Memory research has been guided by two powerful metaphors: the storehouse (computer) and the correspondence metaphor. The latter emphasizes the dependability of retrieved mnemonic information and draws upon ideas about the state dependency and reconstructive character of episodic memory. We used a new movie to unveil the neural correlates connected with retrieval, monitoring, and control processes, and memory accuracy (MAC), according to the paradigm of Koriat and Goldsmith (1996a,b). During functional magnetic resonance imaging, subjects performed a memory task which required (after an initial learning phase) rating true and false statements [retrieval phase (RP)], making confidence judgments in the respective statement [monitoring phase (MP)], and deciding for either venturing (volunteering) the respective answer or withholding the response [control phase (CP)]. Imaging data pointed to common and unique neural correlates. Activations in brain regions related to RP and MAC were observed in the precuneus, middle temporal gyrus, and left hippocampus. MP was associated with activation in the left anterior and posterior cingulate cortex along with bilateral medial temporal regions. If an answer was volunteered (as opposed to being withheld) during the CP, temporal, and frontal as well as middle and posterior cingulate areas and the precuneus revealed activations. Increased bilateral hippocampal activity was found during withholding compared to volunteering answers. The left caudate activation detected during withholding compared to venturing an answer supports the involvement of the left caudate in inhibiting unwanted responses. Contrary to expectations, we did not evidence prefrontal activations during withholding (as opposed to volunteering) answers. This may reflect our design specifications, but alternative interpretations are put forth.

## Introduction

When considering the contribution of subject-controlled processes to memory performance, it is important to distinguish between two different properties of memory: quantity and accuracy (Klatzky and Erdelyi, 1985). Koriat and Goldsmith (1994, 1996a) and Herrmann et al. (1996) have shown that these two features have received rather different emphasis in current research practices. With the quantity-oriented and accuracy-oriented approaches to memory, two fundamentally different ways of thinking about memory have been introduced. These ways map onto the distinction between two memory metaphors, the storehouse (where memory is seen as a storehouse garnering items for a later retrieval and is therefore defined in terms of the number of items that can be recovered; Gruneberg and Morris, 1978; Roediger, 1980; Markowitsch, 1994, 2008) and the correspondence metaphor (that construes memory in terms of its capability to faithfully represent past events, rather than just in terms of the quantity of items that are remembered and therefore are remaining in store) (Koriat and Goldsmith, 1996b).

According to Koriat and Goldsmith (1996b), experimental, laboratory memory research is preponderantly quantity-oriented, while in everyday-life the importance of the accuracy-oriented framework is underscored. A common example that illustrates the difference between the two approaches pertains to eyewitness reports: according to the quantity-oriented approach it would be important how much information about an offender can be retrieved, while the accuracy-oriented framework concerns the question whether essential information (e.g., the facial features of an offender) can be remembered. Moreover accuracy measures assess executive components of memory control by evaluating the correctness of retrieved information, and whether certain information would be reported if someone had for example to act as a witness in court (Kelley and Sahakyan, 2003).

**Figure 1:**
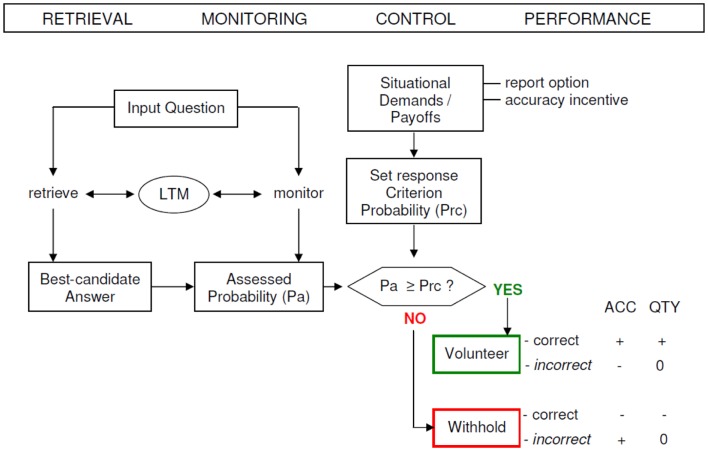
**A schematic model of the strategic regulation of memory accuracy and memory quantity performance**. Performance effects are signified by plus (+ = increase), minus (− = decrease), and zero (0 = no effect). LTM, long-term memory; ACC, accuracy; QTY, quantity; Pa, assessed probability; Prc, response criterion probability (modified from Koriat and Goldsmith, 1996b).

The paradigm of Koriat and Goldsmith enables a separated evaluation of quantity and accuracy. Memory quantity performance is defined as the input-bound percentage of statements that were correctly answered (e.g., conditional on the number of input items), whereas memory accuracy (MAC) performance is formalized as the output-bound percentage of statements that were correct (e.g., conditional on the number of output items). The output-bound accuracy measures uniquely reflect the dependability of the reported information, that is, the extent to which each reported item can be counted on to be correct (Goldsmith et al., 2002). MAC performance is tied to the individual competence of controlling the correctness of given answers and deciding to volunteer correct answers and withhold incorrect answers, respectively (see Figure 1).

The paradigm of Koriat and Goldsmith entails three different phases of recall in which different monitoring processes proceed. In the retrieval phase (RP) subjects are presented with a set of memory questions and are requested to answer each of them, even if they have to guess. However, this phase is executed under forced recall conditions and quantity as well as accuracy performance is equalized in the RP.

In the monitoring phase (MP) the monitoring process is activated, hence subjects are required to rate their confidence of whether the retrieved item is correct or not (0–100%). In the control phase (CP) subjects are free to decide whether to bet on the correctness of their answer or not (volunteering or withholding). The control mechanism operates by establishing a confidence threshold (response criterion) on the monitoring output: if the assessed probability of being correct passes the threshold, the answer is volunteered; otherwise the answer is withheld. The setting of the threshold is sensitive to the gain for giving a correct answer relative to the cost of delivering an incorrect response.

The impact of monitoring and control on (free-report) memory performance has been proven to depend on several elements, such as the monitoring effectiveness, the control sensitivity, and the response criterion setting (Koriat and Goldsmith, 1996b). The extent to which the assessed probabilities successfully discern correct from wrong candidate answers and weighing the relative payoffs for accuracy and quantity for coming up with an optimal criterion level are captured by the construct of monitoring effectiveness.

The control sensitivity is the degree to which volunteering or withholding of answers is in fact susceptible to the monitoring output response. The (report) criterion setting reveals the probability level that is appointed (set) in consonance with the incentive to be accurate (and contending demands for quantity and accuracy). The criterion setting or report control policy can be gaged as “the cut-off on each participant’s assessed-probability-correct ratings that best predicts his or her actual volunteering-withholding decisions in the free-report phase” (Halamish et al., 2012, p. 2). These three factors strongly influence quantity and accuracy of memory performance and therefore their investigation is warranted when examining MAC (Goldsmith et al., 2002).

The aim of the current study is to implement the model of the strategic regulation of MAC and memory quantity performance by Koriat and Goldsmith into a functional magnetic resonance imaging (fMRI) design, in order to disentangle the neural correlates connected with the three main processes: retrieval, monitoring, and control and, additionally unravel the brain areas related to MAC performance. For the purpose of approximating real-life conditions, we used in the present study a short film with emotional material. During the scanning procedure, subjects had to respond to veridical and incorrect statements pertaining to the content of the movie. This format shares a similitude to the study of Mendelsohn et al. (2010): during the videotape audiovisual material was presented, while the fMRI-scanning phase contained only written statements. These statements had not been presented previously and therefore it is assumed that they “could not be answered properly without mentally reconstructing studied material” (Mendelsohn et al., 2010, p. 1). By employing this design we had the goal to come close to eyewitness testimony circumstances, where the testifiers might have watched a criminal event and subsequently, when they appear in court, they have to respond verbally.

## Materials and Methods

### Participants

Twenty-nine subjects [14 male (mean age = 26, SD = 2.8, min = 22, max = 31 years), 15 female (mean age = 24.13, SD = 3.4, min = 20, max = 30 years)] without prior history of psychiatric conditions (including gambling problems) or neurological diseases (as determined by thorough screening) and with normal or corrected to normal vision participated in the experiment. The study was approved by the local ethics committee. Female and male subjects did not significantly differ with respect to their mean age (female versus male, *T* = 0.55, *p* = 0.592). All participants (mean age = 25, SD = 3.2, min = 20, max = 31 years) were right-handed, as assessed by the Edinburgh inventory (Oldfield, 1971), and native speakers of German. Participants were recruited from the University of Bielefeld community. Written consent for taking part in the study and publication of study’s data in an anonymized form was obtained from all the participants. The participants received course credits or 20 Euros (plus the bonus they gained for correctly volunteering during the scanning procedure).

#### Neuropsychological tests

Participants underwent neuropsychological testing including standard assessments of intelligence, working memory, long-term explicit memory, visuo-constructive abilities, executive functioning, and attention. Intelligence was evaluated with the LPS (Leistungsprüfsystem)-4 (Horn, 1983), handedness, with the Edinburgh Handedness Inventory (Oldfield, 1971) and attention and concentration, with the d2-Test (Brickenkamp and Zillmer, 1998), the Trail Making Test A (Lezak, 1995), and the forward digit span subtest of the Wechsler-Memory Scale-Revised (Härting et al., 2000). Anterograde explicit memory was tested with the Verbal Learning and Memory Test (Helmstaedter et al., 2001) and the Rey–Osterrieth Complex Figure (Lezak, 1995) and working memory was evaluated with the backward digit span subtest of the Wechsler-Memory Scale-Revised (Härting et al., 2000). Executive functions and decision-making were examined with the Game of Dice task (Brand et al., 2005; Brand and Markowitsch, 2010) and the Trail Making Test B (Lezak, 1995). Trail Making Test B served also for testing cognitive flexibility. Verbal fluency tasks (animal words and words starting with the letters F, A, and S) (Lezak, 1995) were given to test word fluency as well as executive functions. In order to aid the exclusion of subjects with personality or psychiatric problems, three scales were given to the participants for self administration, such as the Beck Depression Inventory (BDI; Beck et al., 1995), the Freiburg Personality Inventory (FPI; Fahrenberg et al., 2001), and the Brief Symptom Inventory (BSI; Derogatis, 1993).

### Stimuli

#### Videotape

A short film named “The New Cat,” with an approximate duration of 6 min and emotional material appropriate for students was shown on a computer screen that was 13″ in size. For a consistent and sufficing volume external boxes were used. The film was shown about 2 h prior to the scanning phase. This film by Ziv Shachar (originally entitled “GATO-NOVO”) was identified as being an adequate stimulus material for the research project “The assessment of eyewitness memory: a multi componential, correspondence oriented approach” by research colleagues from Haifa University (Koriat et al., 2000), who obtained the rights to utilize it for research purposes and who used it for behavioral, but not for neuroimaging experiments within the common project financed by the European Commission (De Mulder et al., 2010). (The design resembles that of Pansky and Tenenboim, 2011, though in that study a 6.5 min slide show had been used instead of a film). The soundtrack of the film (originally in Hebrew) was translated to German and English, respectively, to allow its use by the German and English research EU partners. The film has a good range of scenic details, well suited for the fMRI procedure. The movie is about a young adult man who is fond of dogs, but has problems with keeping them in the house, because they make dirt. He decides to have a cat as a pet, but he gets shortly in trouble with this, because he treats the cat like he would treat a dog. After only 1 day, the cat jumps from the window sill and is run over by a car driven by a young woman. Later the young woman and the main character fall in love with each other and they bury together the cat. The pair then moves to live together; the woman gives a dog as a present to the young man. None of the participants in the study indicated having seen the video before. Furthermore subjects were not communicated that their memory of the movie would be probed later (“incidental” encoding condition).

#### Statements

About 180 statements concerning the story of the film were constructed, of which one half concerned true details and the other half contained incorrect details of the film. Moreover, all true and all false statements were consistently related to different categories, like content, perception, and action. The formulated statements were short (with a range between 5 and 10 words in the German language version), in order to improve their readability on the screen inside the scanner. We attempted to have approximately similar numbers of items for the different categories and also to match the statements with respect to difficulty or complexity (see Appendix).

#### Experimental tasks

**Figure 2:**
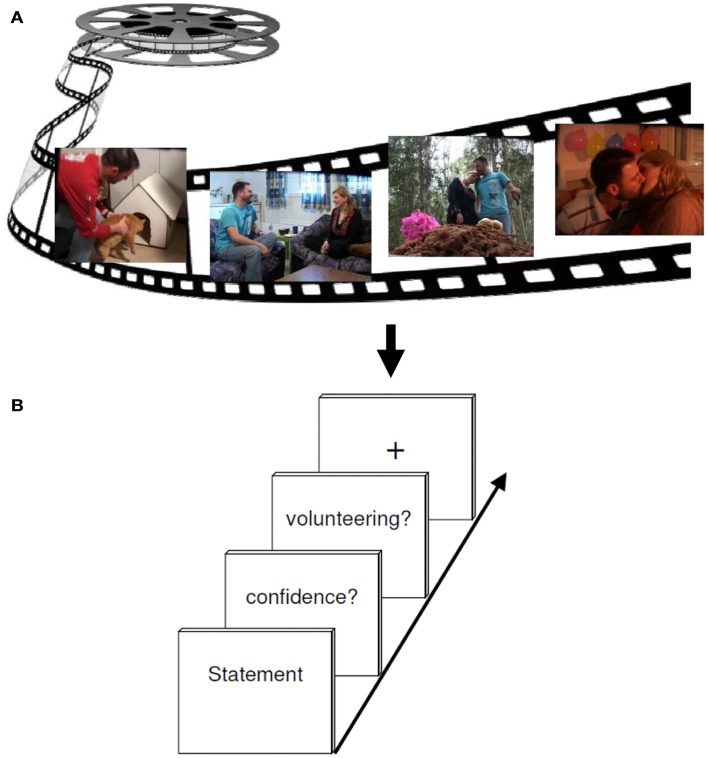
**Experimental design**. **(A)** The encoding session consisted of watching a 6 min emotional film in a quiet room. **(B)** About 2 h later, participants performed a test session while undergoing an fMRI brain scan. Each retrieval task trial included either a correct or incorrect statement regarding events in the movie, to which subjects had to responded either “yes” (true) or “no” (false) (RETRIEVAL phase). Subsequently, participants had to rate their confidence (MONITORING phase) about the foregoing response (high versus low secureness) and had to decide either to volunteer or to withhold their answer (CONTROL phase).

The tasks used during event-related fMRI procedure required the subjects to evaluate correct and incorrect statements from the videotape they saw before. In sum 180 statements appeared on a computer screen in a random order. Each statement had to be assessed with respect to its quality as being true or false (RP). This was followed by a confidence rating offering the options of three increments, namely 100% confidence, 75% confidence, and 50% confidence (MP). For further analyses we decided to differentiate between high (100%) and low confidence (combining 50 and 75%). In the next step, subjects had to decide whether to volunteer (bet) or withhold the answer (CP). Volunteering consisted of expressing the will to bet on the correctness of the given answer. An explicit bonus system of moderate incentive was implemented to motivate accurate responding. All participants were recompensed according to the number of bonus points. If a correct answer was volunteered one bonus point could be earned; if an incorrect answer was volunteered, one bonus point could be lost. When deciding for withholding the response, no bonus points were granted or deducted, irrespective of the correctness of the answer. For the baseline a fixation cross was presented to complete the foregone sequence and draw attention to the next sequence (see Figure 2).

During the fMRI measurement, subjects made their choices using three fingers of their right hand (index finger, middle finger, and ring finger) on a three button response device. Random jitter was included to prevent correlation of event regressors. For this, statements were presented between 3 and 5 s, and the confidence retrieval, the question for volunteering/withholding as well as the fixation cross were illustrated between 2 and 3 s. The whole run took about 43 min. In order to prevent head movements throughout the scan, the experiment was divided into two consecutive scans, each containing one half of the statements. Each run lasted an average of 17 min. For stimulus presentation and response collection, the software Presentation 9.0^1^ (Neurobehavioral Systems, Albany, CA, USA) was used. During the fMRI experiment, the stimulus display was back-projected onto a screen mounted on a custom head coil.

#### Pre-scanning procedure

In order to get familiarized with the experimental set-up, subjects took part in a presentation of neutral statements adapted for utilization inside the scanner. Some of these 45 statements were true and some of them were false, e.g., “elephants have thick skin.” Subjects completed the pre-testing-learning phase on the same PC the videotape was shown earlier and used the numbers “1” “2” and “3” of the PC keyboard. It was important that subjects deliberated on the statements, in order to get used to the available time slot. The instruction was similar to the fMRI-scanning procedure. The goal of the pre-scanning procedure was to make sure that subjects internalize the instruction and automated button-pushing, in order to secure an accurate scanning procedure. All participants reached the required cut-off value, which was saved in a txt file and therefore made available for an evaluation. For stimulus presentation and response collection, the software Presentation 9.0 (Neurobehavioral Systems, Albany, CA, USA; see text footnote 1) was utilized.

#### MR technical parameters

Functional MR images were acquired on a Siemens Magnetom Investigational Device 7T syngo MR B15 with echo planar imaging (EPI) capability. Head motion was restricted using expandable foam pads that surrounded the head. Stimuli were presented on a screen. Multislice T2*-weighted echo planar images were achieved from a gradient-echo sequence with the following parameters: repetition time (TR) = 3000 ms, echo time (TE) = 29 ms, field of view (FOV) = 230 mm, flip angle = 76°, slice thickness = 4 mm. About 30 axial slices were oriented in the plane of the anterior-posterior commissure and covered the whole brain. For each subject, additional high-resolution anatomical images were acquired using the 3D T1-weighted magnetization prepared, rapid acquisition gradient echo (MP-RAGE) sequence with the parameters: TR = 2300 ms, TE = 3.93 ms, inversion time (TI) = 1100 ms, flip angle = 12°, FOV = 256 × 256 mm, matrix size = 1.0 mm × 1.0 mm × 1.0 mm, 160 sagittal slices with a thickness of 1 mm (Poser and Norris, 2009; Poser et al., 2010).

#### Image processing and data analysis

Functional volumes were analyzed with SPM5^2^ (Wellcome Department of Imaging Neuroscience, London, UK) implemented in MATLAB 7 (The Mathworks Inc., Natick, MA, USA). The images were realigned, normalized into the Montreal Neurological Institute (MNI) coordinate space and smoothed with a 5 mm × 5 mm × 5 mm Gaussian kernel (full width half maximum).

Parameter estimates of the resulting general linear model were calculated for each subject and each voxel. For population inference, a second level analysis was performed, using the contrast estimates for the simple effect of each experimental condition.

Differential contrasts of interest were calculated according to the experimental factors RP (correct answer versus incorrect answer, and vice versa), MP (high confidence versus low confidence, and vice versa), and CP (volunteering versus withholding, and vice versa) as well as RP versus MP (and vice versa), RP versus CP (and vice versa), and MP versus CP (and vice versa) to assess differential modulation of the BOLD signal induced by each factor. To detect only MAC (without an overlap to quality) according to the model of Koriat and Goldsmith (see Figure 1) the factor MAC was calculated: MAC+ (withholding an incorrect answer leads to an increase in MAC) versus MAC− (volunteering an incorrect answer leads to a decrease in MAC) (and vice versa).

The statistical threshold for within- and between-group comparisons was set to *p* < 0.001, corrected for multiple comparisons at the cluster level. This threshold was required due to the combination of a high Tesla Scanner and a rather complex experimental design.

#### Localization of activations

SPM_T_ maps resulting from the group analysis were superimposed onto a group mean MR image which was calculated from the normalized anatomical T1-images of each subject (see above). Standard stereotactic coordinates of voxels showing local maximum activation were determined within areas of significant relative changes in neural activity associated with different experimental conditions. Maxima were anatomically localized and labeled with an anatomical SPM5 toolbox, namely AAL, which refers to the Automated Anatomical Labeling map which is a three-dimensional map containing 116 brain regions co-registered to standard MNI space. MNI coordinates refers to a standard brain imaging coordinate system developed by the MNI (Tzourio-Mazoyer et al., 2002).

## Results

### Behavioral data

#### Neuropsychological testing

Individual neuropsychological data were within the range of reference population norms for all tests which were administered.

#### Responding behavior during scanning

For the differential contrasts defined in the fMRI experiment *T*-tests for paired samples were executed to analyze subjects’ response behavior. *T*-tests revealed that subjects responded to the statements mainly correctly (RP: correct answer versus incorrect answer, *T* = 23.59, *p* < 0.001). During retrieval process, different responding pattern can be distinguished: (A) to assume a true statement (hit), (B) to decline a true statement (miss), (C) to decline a false statement (correct rejection), and (D) to assume a false statement (false alarm). Correct responding is therefore defined as either correct rejection or hit, in contrast to incorrect responding that represents either false alarm or miss. The results of the present study show that when a statement was answered correctly this was a consequence of correct rejection significantly more often than it was resulting from a hit (correct rejection versus hit, *T* = 6.56, *p* < 0.001). When a statement was answered incorrectly this was because of a miss significantly more often than it was due to a false alarm (miss versus false alarm, *T* = 8.57, *p* < 0.001).

**Figure 3:**
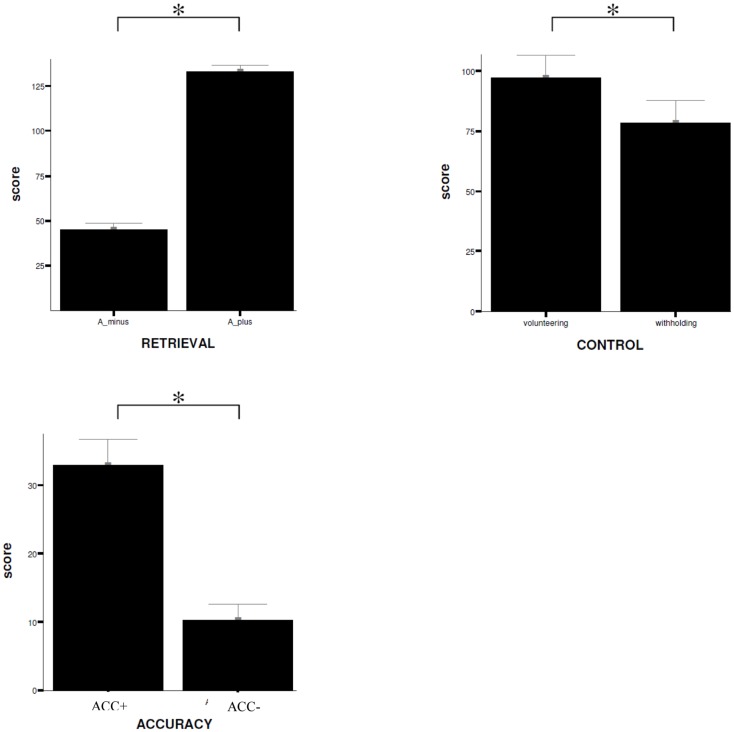
**Behavioral data**. Comparison of mean ratings (±SD) given by the subjects on items during scanning procedure for the different experimental conditions, RETRIEVAL, CONTROL, and ACCURACY. W+, volunteering; W−, withholding; SD, standard deviation; *trend of statistical significance (*p* < 0.05, uncorrected). A_ minus = incorrect answering; A_ plus = correct answering.

No significant difference can be reported for the confidence rating of the statements (MP: high confidence versus low confidence, *T* = −1.43, *p* = 0.16). Moreover, the analyses reveal that subjects rather volunteered an answer instead of withholding it (CP: volunteering versus withholding, *T* = 2.1, *p* < 0.05). Participants show respectively a significant increase in MAC (MAC+ versus MAC−, *T* = 9.18, *p* < 0.001). Data are displayed in Figure 3.

### fMRI data

#### Retrieval process (correct versus incorrect responding)

**Figure 4:**
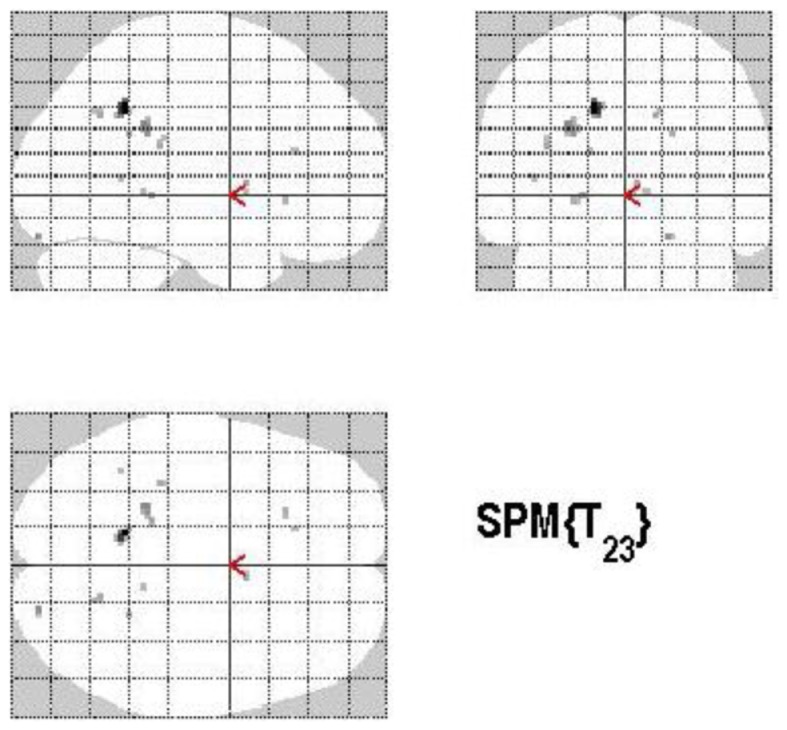
**Group activations for the contrast between correct versus incorrect answers (RETRIEVAL)**. Activations are superimposed on the anatomical group mean image (see Materials and Methods), depicting statistically significant relative increases in neural activity at *p* < 0.001, uncorrected. See Table 1 for the exact MNI coordinates.

The main effect of the correct relative to incorrect (A+ > A−) answers revealed significant differential bilateral activations of the precuneus and activations of the left hippocampus, the left insula, left middle temporal gyrus (MTG), and right lingual gyrus (*p* < 0.001, uncorrected). The reverse contrast (A− > A+) did not show any differential activation. Data are displayed in Table 1 and Figure 4.

**Table 1:** **Group activations for the contrast between correct versus incorrect answers (RP), *p* < 0.001, uncorrected**.

Anatomical region	Side	MNI coordinates**		
		*x*	*y*	*z*
**CORRECT > INCORRECT**				
Precuneus	L	−14	−48	40
	R	14	−16	36
Hippocampus	L	−20	−36	0
Insula	L	−36	−32	22
Lingual gyrus	R	10	−40	2
Middle temporal gyrus	L	−42	−50	8

Brain regions within the boundaries of the AAL* atlas.

*AAL refers to the Automated Anatomical Labeling map which is a three-dimensional map containing 116 brain regions co-registered to standard MNI space.

**MNI coordinates refers to a standard brain imaging coordinate system developed by the Montreal Neurological Institute.

#### Monitoring process (high confidence versus low confidence)

**Figure 5:**
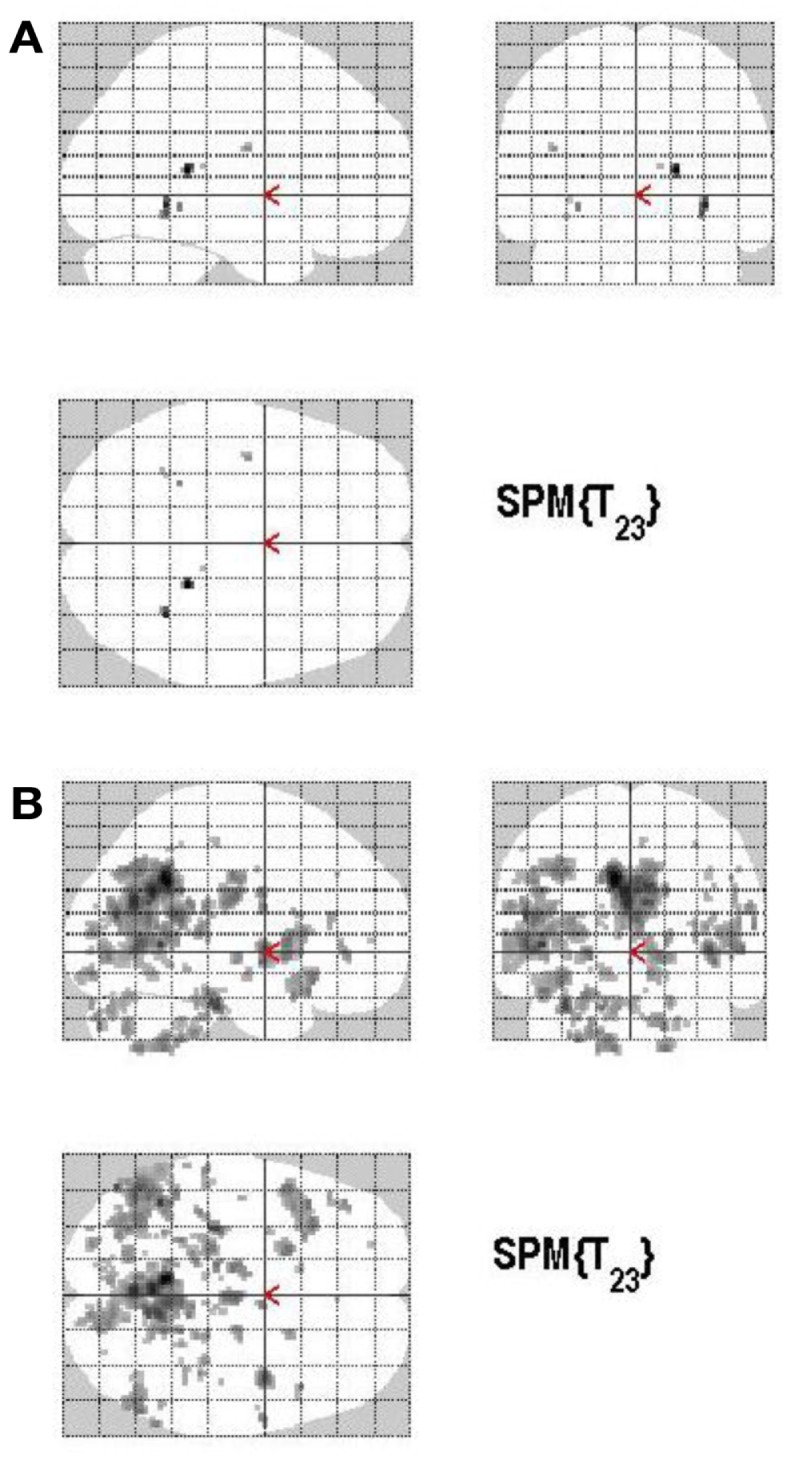
**(A)** Group activations for the contrast between high versus low secureness (MONITORING). Activations are superimposed on the anatomical group mean image (see Materials and Methods), depicting statistically significant relative increases in neural activity at *p* < 0.001, uncorrected. See Table 2A for the exact MNI coordinates. **(B)** Group activations for the contrast between low versus high secureness (MONITORING). Activations are superimposed on the anatomical group mean image (see Materials and Methods), depicting statistically significant relative increases in neural activity at *p* < 0.001, uncorrected. See Table 2B for the exact MNI coordinates.

Areas of significant differential activation revealed by high confidence relative to low confidence (S+ > S−) ratings were located bilaterally in the fusiform gyrus and the left lingual gyrus and the left parahippocampal gyrus (*p* < 0.001, uncorrected) (Table 2A; Figure 5A). The reverse contrast (S− > S+) demonstrated, amongst others, bilateral activation of the hippocampus, the angular gyrus, precentral gyrus, lingual, middle occipital, inferior parietal and postcentral gyri, putamen, Rolandic operculum, different temporal and frontal and occipital regions, the left precuneus, and the right insula (*p* < 0.05, False Discovery Rate-FDR corrected); at *p* < 0.05, Family Wise Error (FWE) corrected the left precuneus was activated. See Table 2B and Figure 5B for detailed information.

**Table 2:** **Group activations for the contrast between high versus low confidence (MP), *p* < 0.001, uncorrected**.

Anatomical region	Side	MNI coordinates		
		*x*	*y*	*z*
**(A) HIGH CONFIDENCE > LOW CONFIDENCE**				
Fusiform gyrus	R	34	−48	−4
	L	−34	−50	−10
Parahippocampal area	L	−28	−42	−6
Lingual gyrus	L	−32	−48	−2
**(B) LOW CONFIDENCE > HIGH CONFIDENCE**				
Precuneus	L	−8	−50	36
	L	−2	−56	30
	L	−2	−64	24
Cuneus	L	−2	−80	28
	R	12	−96	10
Middle temporal gyrus	L	−44	−52	4
	L	−52	−62	4
	L	−52	−42	10
	R	56	−62	2
	R	54	−68	14
	R	62	−54	12
	R	52	−52	10
	R	60	−50	0
	R	58	−36	−8
Superior temporal pole	L	−44	22	−18
	L	−54	12	−8
Superior temporal gyrus	R	62	−50	20
	L	−60	−30	22
	L	−52	−34	10
	R	38	−32	12
	R	56	−44	20
Inferior temporal gyrus	R	58	−60	−6
Fusiform gyrus	L	−32	−24	−26
Insula	R	40	0	0
	R	44	18	0
	R	36	−12	22
	R	36	−18	4
Middle occipital gyrus	R	52	−68	26
	R	30	−72	22
	R	38	−72	16
	R	36	−84	30
	L	−24	−86	10
	L	−32	−88	14
Superior occipital gyrus	L	−14	−84	22
Inferior occipital gyrus	R	30	−86	−16
Parahippocampal area	L	−22	−26	−20
Hippocampus	R	24	−20	−16
	L	−30	−6	−26
Putamen	L	−22	12	0
	L	−30	2	−2
	R	22	14	−2
Inferior frontal gyrus pars opercularis	L	−52	12	6
	L	−34	24	−12
	L	−52	14	20
	L	−58	12	16
Inferior frontal gyrus pars triangularis	L	−44	40	0
	L	−46	32	16
Superior frontal gyrus medial	L	2	36	38
Superior frontal gyrus	L	−24	54	6
Middle frontal gyrus	L	−38	24	40
Supramarginal gyrus	L	−48	−44	34
	L	−56	−52	34
	L	−52	−30	24
Postcentral gyrus	R	−40	−32	10
Lingual gyrus	L	−14	−56	−10
	L	−16	−68	−6
	R	14	−60	−8
Precentral gyrus	R	54	−4	40
	L	−42	−2	30
Postcentral gyrus	L	−46	−12	38
	R	58	−22	42
	R	54	−16	40
Posterior cingulate cortex	L	−6	−44	8
Middle cingulate cortex	R	6	−16	34
	R	10	−30	32
Calcarine sulcus	L	−12	−80	12
	L	−14	−66	4
	L	−14	−72	8
Inferior parietal gyrus	L	−54	−48	50
	L	−46	−58	46
	L	−42	−56	48
	R	32	−42	52
	R	58	−48	40
Rolandic operculum	R	50	−18	12
	R	52	−10	18
	R	46	−12	20
	L	−60	−6	12
Angular gyrus	R	56	−48	30
	R	50	−66	40
	L	−46	−58	34
Paracentral lobule	R	16	−42	50

Brain regions within the boundaries of the AAL* atlas.

#### Control process (volunteering versus withholding)

**Figure 6:**
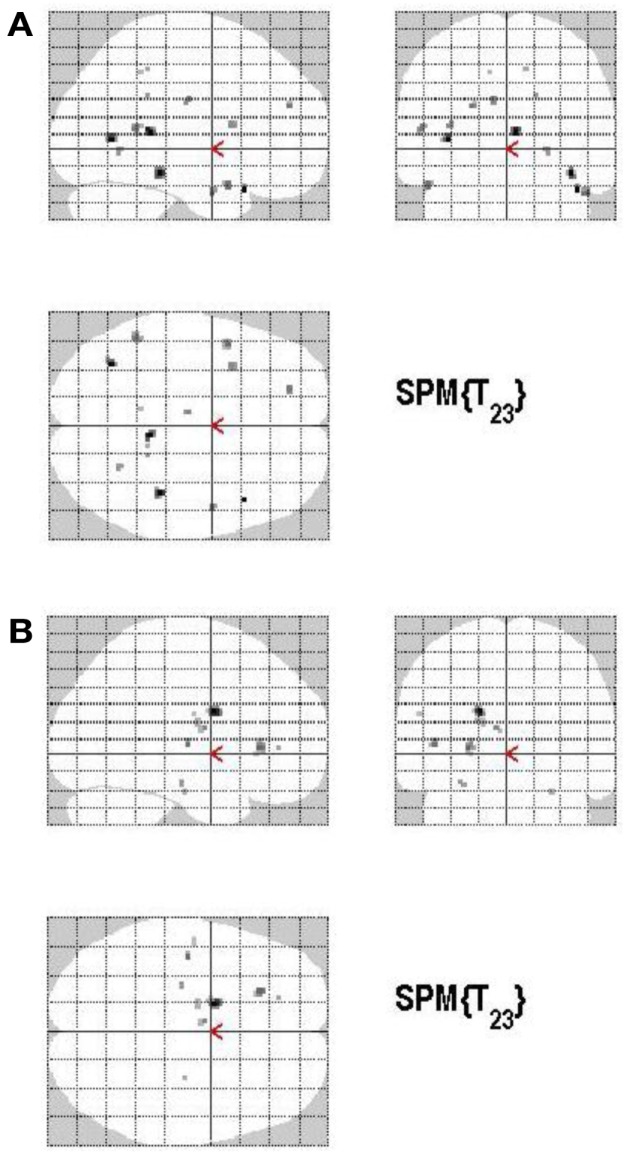
**(A)** Group activations for the contrast between volunteering versus withholding (CONTROL). Activations are superimposed on the anatomical group mean image (see Materials and Methods), depicting statistically significant relative increases in neural activity at *p* < 0.001, uncorrected. See Table 3A for the exact MNI coordinates. **(B)** Group activations for the contrast between withholding versus volunteering (CONTROL). Activations are superimposed on the anatomical group mean image (see Materials and Methods), depicting statistically significant relative increases in neural activity at *p* < 0.001, uncorrected. See Table 3B for the exact MNI coordinates.

Volunteering relative to withholding (W+ > W−) produced bilateral activations of temporal, frontal, and cingulate regions, namely of the MTG, the superior temporal pole, the left middle frontal and left inferior frontal cortex (pars opercularis), the left precuneus and the right posterior cingulate cortex (*p* < 0.001, uncorrected) (see Table 3A; Figure 6A). The reverse contrast, namely, withholding (W− > W+), revealed bilateral activation of the hippocampus, the left caudate nucleus, the left Heschl region, and the left postcentral gyrus (*p* < 0.001, uncorrected). Data are presented in Table 3B and Figure 6B.

**Table 3:** **(A,B) Group activations for the contrast between volunteering versus withholding (CP), *p* < 0.001, uncorrected**.

Anatomical region	Side	MNI coordinates		
		*x*	*y*	*z*
**(A) VOLUNTEERING > WITHHOLDING**				
Superior temporal pole	R	44	20	−24
	L	−48	10	−22
Middle temporal gyrus	R	50	0	−26
	L	−52	−46	12
Posterior cingulate cortex	R	6	−38	10
Middle cingulate cortex	R	12	−40	48
Middle frontal gyrus	L	−22	48	26
Inferior frontal gyrus pars opercularis	L	−36	12	14
Precuneus	L	−10	−44	46
**(B) WITHHOLDING > VOLUNTEERING**				
Heschl region	L	−44	−14	6
Hippocampus	L	−28	−18	−16
	R	28	−16	−22
Caudate nucleus	L	−14	−8	18
Postcentral gyrus	L	−54	−10	24

Brain regions within the boundaries of the AAL* atlas.

#### Monitoring versus retrieval process

**Figure 7:**
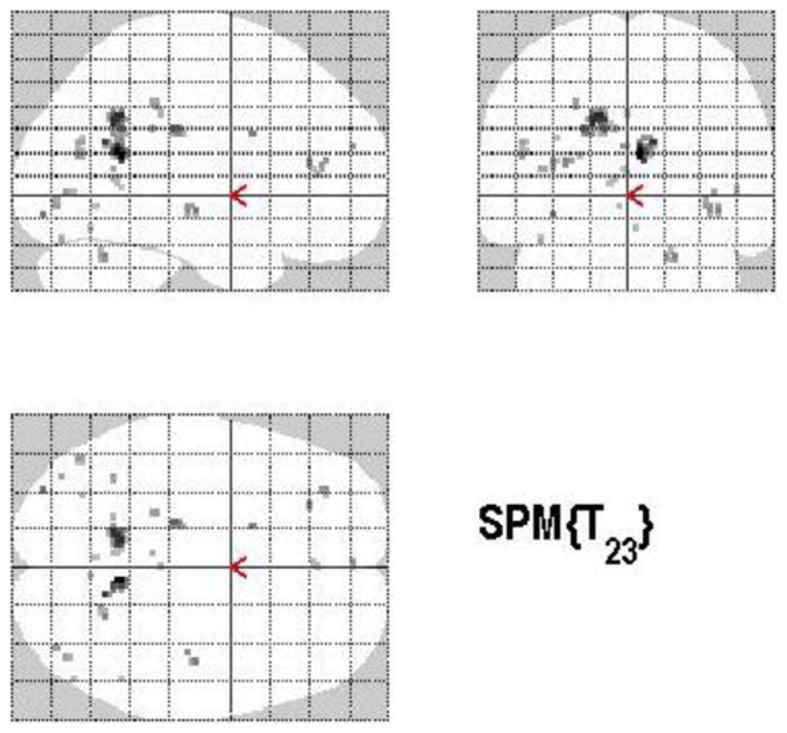
**Group activations for the contrast between MONITORING versus RETRIEVAL**. Activations are superimposed on the anatomical group mean image (see Materials and Methods), depicting statistically significant relative increases in neural activity at *p* < 0.001, uncorrected. See Table 4 for the exact MNI coordinates.

The main effect of monitoring relative to retrieval revealed significant differential bilateral activations of the inferior occipital gyrus, precuneus, MTG, left middle cingulate cortex, left anterior and posterior cingulate cortex, left middle and superior frontal gyri (*p* < 0.001, uncorrected) (Table 4; Figure 7). The reverse contrast, retrieval (relative to monitoring), did not reach statistical significant activation.

**Table 4:** **Group activations for the contrast between MP versus RP, *p* < 0.001, uncorrected**.

Anatomical region	Side	MNI coordinates		
		*x*	*y*	*z*
**MP > RP**				
Precuneus	R	6	−52	20
	R	12	−58	24
	L	−6	−54	8
Middle cingulate cortex	L	−12	−50	36
	L	0	−32	36
Posterior cingulate cortex	L	−6	−36	30
Anterior cingulate gyrus	L	0	40	10
	L	−2	38	12
Inferior occipital gyrus	L	−34	−86	−8
	R	36	−80	−4
Middle occipital gyrus	R	40	−76	2
Middle frontal gyrus	L	−34	44	16
Superior frontal gyrus medial	L	−2	56	22
Middle temporal gyrus	L	−48	−70	18
	L	−40	−54	12
	R	50	−72	2
	R	50	−50	4

Brain regions within the boundaries of the AAL* atlas.

#### Control versus retrieval

**Figure 8:**
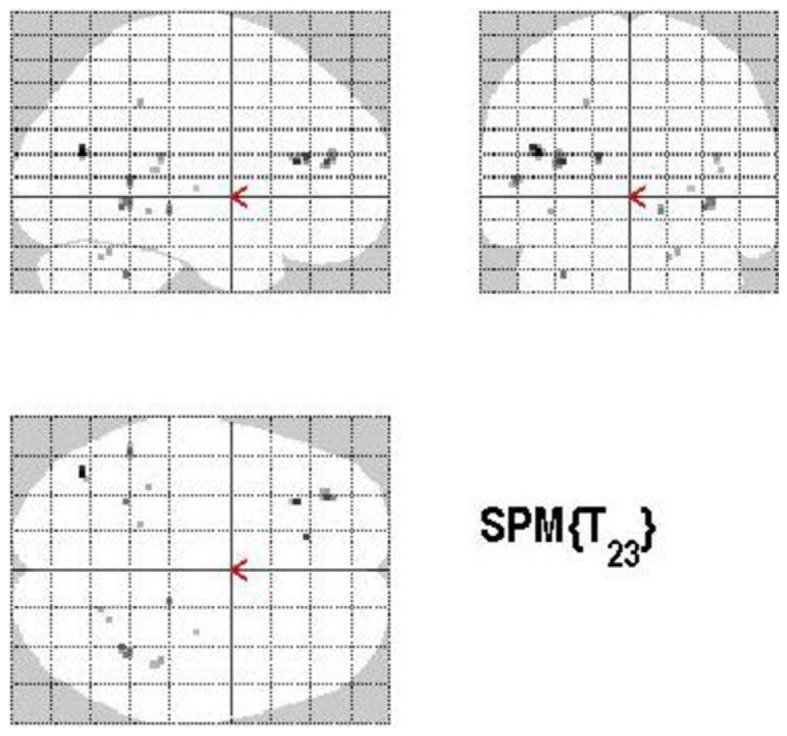
**Group activations for the contrast between CONTROL versus RETRIEVAL**. Activations are superimposed on the anatomical group mean image (see Materials and Methods), depicting statistically significant relative increases in neural activity at *p* < 0.001, uncorrected. See Table 5 for the exact MNI coordinates.

Areas of significant differential activation revealed by control relative to retrieval were located in the left middle frontal gyrus, left MTG, right fusiform gyrus, right putamen, right Rolandic operculum, and the right superior temporal gyrus (STG) (*p* < 0.001, uncorrected) (See Table 5; Figure 8). Again, the reverse contrast, retrieval (relative to control) did not show any differential activation.

**Table 5:** **Group activations for the contrast between CP versus RP, *p* < 0.001, uncorrected**.

Anatomical region	Side	MNI coordinates		
		*x*	*y*	*z*
**CP > RP**				
Middle temporal gyrus	L	−42	−68	20
	L	−52	−46	8
Superior temporal gyrus	R	42	−34	12
Middle frontal gyrus	L	−32	44	16
Fusiform gyrus	R	34	−50	−4
Rolandic operculum	R	40	−32	18
Putamen	R	28	−16	4

Brain regions within the boundaries of the AAL* atlas.

#### Monitoring versus control process

**Figure 9:**
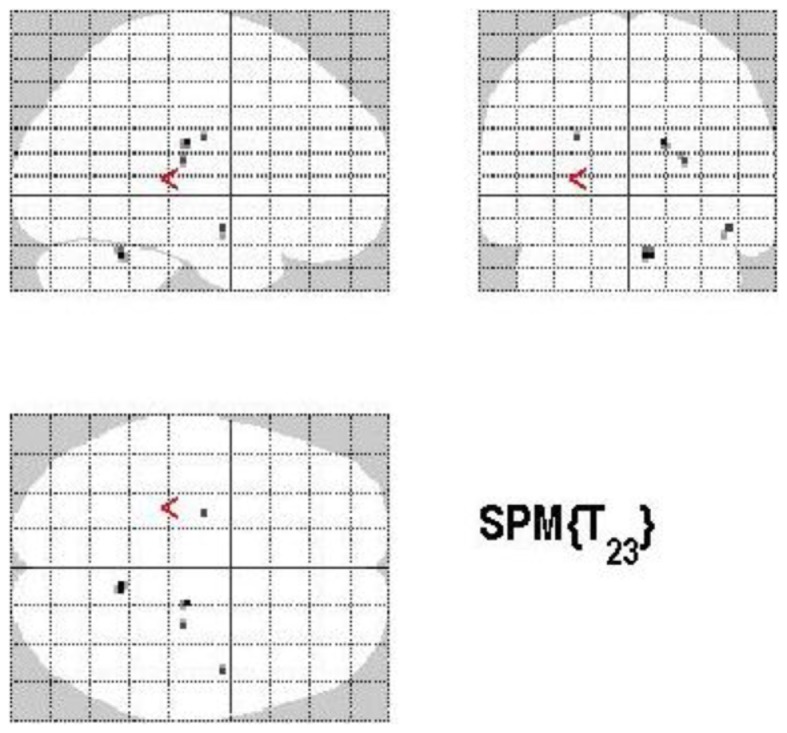
**Group activations for the contrast between MONITORING versus CONTROL**. Activations are superimposed on the anatomical group mean image (see Materials and Methods), depicting statistically significant relative increases in neural activity at *p* < 0.001, uncorrected. See Table 6 for the exact MNI coordinates.

The main effect of monitoring relative to control consisted of significant activations of the right STG (*p* < 0.001, uncorrected) (See Table 6; Figure 9). Due to the fact that only one region reached statistical significance this contrast will not be discussed here. The reverse contrast, control (relative to monitoring), did not reveal any significant activation.

**Table 6:** **Group activations for the contrast between MP versus CP, *p* < 0.001, uncorrected**.

Anatomical region	Side	MNI coordinates		
		*x*	*y*	*z*
**MP > CP**				
Superior temporal gyrus	R	46	−4	−14

Brain regions within the boundaries of the AAL* atlas.

#### Memory accuracy (MAC+ versus MAC−)

**Figure 10:**
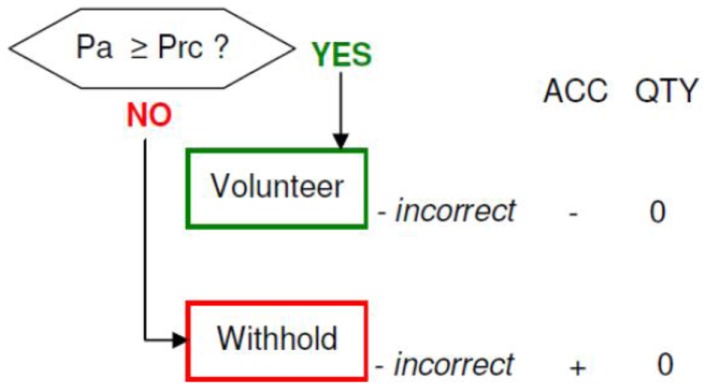
**Memory accuracy and inaccuracy according to the memory paradigm (cf. Figure 1 for abbreviations)**.

**Figure 11:**
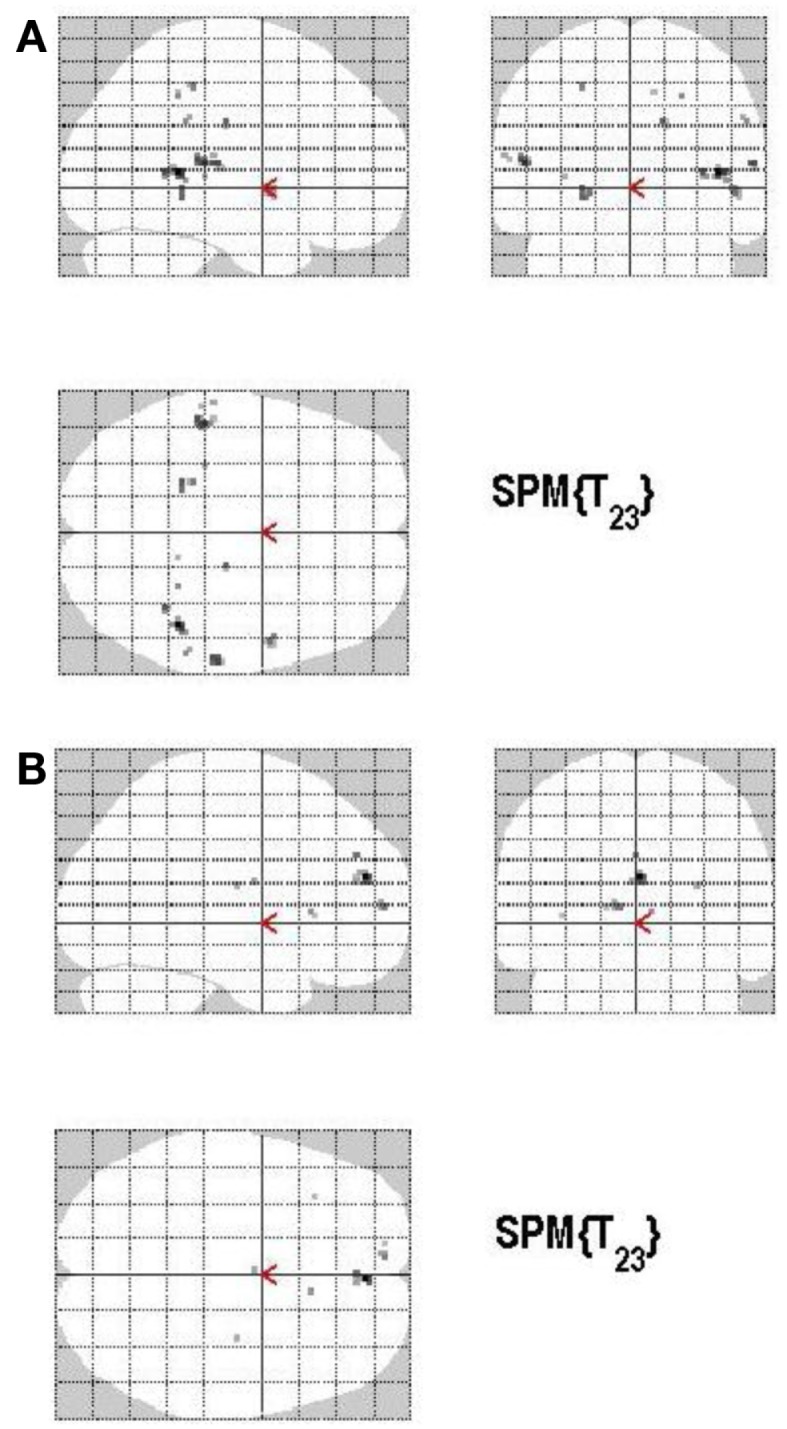
**(A)** Group activations for the contrast between ACC+ versus ACC− (ACCURACY). Activations are superimposed on the anatomical group mean image (see Materials and Methods), depicting statistically significant relative increases in neural activity at *p* < 0.001, uncorrected. See Table 7A for the exact MNI coordinates. **(B)** Group activations for the contrast between ACC− versus ACC+ (ACCURACY). Activations are superimposed on the anatomical group mean image (see Materials and Methods), depicting statistically significant relative increases in neural activity at *p* < 0.001, uncorrected. See Table 7B for the exact MNI coordinates.

There are different ways of defining MAC; in the present study the manner of conceptualizing MAC was influenced by the paradigm put forth by Koriat and Goldsmith (1996a,b). MAC was defined as withholding an *incorrect* answer, whereas memory inaccuracy was related to volunteering an *incorrect answer* (See Figure 10). The main effect of high MAC relative to low MAC revealed significant differential bilateral activations of the STG, the supramarginal gyrus, left hippocampus, left Heschl region, the right superior temporal pole, MTG, and the right precuneus (*p* < 0.001, uncorrected), depicted in Table 7A and Figure 11A. The reverse contrast, low accuracy (relative to high accuracy), revealed activation only of the left hemisphere, namely the insula and the superior frontal gyrus (*p* < 0.001, uncorrected), illustrated in Table 7B and Figure 11B.

**Table 7:** **(A,B) Group activations for the contrast between MAC+ versus MAC− (memory accuracy), *p* < 0.001, uncorrected**.

Anatomical region	Side	MNI coordinates		
		*x*	*y*	*z*
**(A) MAC+ > MAC−**				
Superior temporal gyrus	L	−52	−28	12
	L	−60	−30	14
	R	62	−22	12
Superior temporal pole	R	52	4	−2
Middle temporal gyrus	R	48	−38	4
Hippocampus	L	−24	−40	0
Supramarginal gyrus	R	58	−38	32
	R	56	−36	34
	L	−62	−24	16
Heschl region	L	−32	−28	6
Precuneus	R	12	−42	46
**(B) MAC− > MAC+**				
Superior frontal gyrus medial	L	2	50	22
	L	0	46	32
	L	−8	60	8
Superior frontal gyrus	L	−14	60	8
Insula	L	−36	26	4

Brain regions within the boundaries of the AAL* atlas.

## Discussion

The current study had the goal of unveiling the neural mechanisms connected with retrieval, monitoring, and control processes according to the memory paradigm of Koriat and Goldsmith (1996b) and, furthermore of identifying the neural underpinnings of MAC. Below we discuss the responses of selected regions that were predicted on grounds of previous findings.

### Retrieval process (correct versus incorrect responding)

The factor RP (retrieval; correct versus incorrect responding) induced a significant effect in the behavioral rating during the scanning procedure: subjects gave more correct than incorrect answers, however, this was in general a consequence of correct rejection (instead of a hit). One could argue that these behavioral results resemble a distinctiveness heuristic (Schacter et al., 1999; Gallo et al., 2008; Koriat et al., 2008; McDonough and Gallo, 2012). Gallo (2011) noted that, when participants expect distinctive memories, they seem to be biased to avoid false alarms rather than enhancing true memory decisions (hits). When incorrect answers were given in our study, this resulted basically from a miss (instead of a false alarm). This finding indicates that subjects were able to correctly discriminate between correct and incorrect answers and moreover responded rather cautiously, avoiding risky decisions. Incidentally, no participant showed a tendency for risk-taking behavior on the Game of Dice task (Brand et al., 2005; Brand and Markowitsch, 2010).

When responding correctly instead of giving false (incorrect) answers brain activation was found mainly in areas that are agreed upon to be involved in mnemonic processing. In consonance with other previous studies, we evidenced activation of the left hippocampus during correct responding (in contrast to incorrect answering). In particular, our finding supports the results of a relatively recent study of Mendelsohn et al. (2010) analyzing differential BOLD responses as a function of correctness in the left hippocampus. In this study, young adults saw a documentary movie. A week later they had to accept or reject factual or fictitious verbal statements about the movie while undergoing functional MRI.

The laterality of hippocampus activations during recall and the degree to which hippocampus subserves recollection versus recognition are however topics of ongoing debate (Gilboa et al., 2004, 2006; Addis et al., 2012). Some authors concluded that hippocampus selectively supports recollection, whereas others proposed that hippocampus is equally implicated in familiarity and recollection (Wixted and Squire, 2011; Markowitsch and Staniloiu, 2012). Wixted and Squire (2011) put forth the idea that when Remember and Know judgments are equated for strength at high level, hippocampal activity is elevated to a similar degree for Remember and Know judgments. A recent study however provided findings consistent with the view that hippocampus offers selective support for recollection and fails to respond to adjustments in familiarity strength and does not sustain strength-matched familiarity, which is sustained by perirhinal cortex (Kafkas and Montaldi, 2012). And another new investigation sets the foundation for a compromise, by showing that both dual-process and strength theories are partly correct (Hayes et al., 2011). Ross et al. (2009) proposed that the hippocampus may play a role in disambiguation, which is in sequence organization during recollection. Rutishauser et al. (2008), using single unit recordings, observed that spike activity in hippocampus correlated positively with successful recall of previously perceived stimuli.

With respect to laterality, the left hippocampus was related to verbal memory tasks (Frisk and Milner, 1990). It was also found to facilitate general coherence of an episode or a scene and play a role in self projection of oneself in comparison to another. Additionally it was linked to context dependant recall of episodic information and vividness of details (Viard et al., 2012). The age of participants can also affect the laterality, with older people showing greater right hippocampus activation or bihemispheric activation during recall of episodic information than younger people (Oddo et al., 2010).

An interesting finding however comes from a recent review that showed that specific cues (verbal) associated to *strictly* episodic events elicited higher left (posterior) hippocampal activation than episodic events triggered by specific cues (Oddo et al., 2010; but see also Addis et al., 2012; Viard et al., 2012). This report lends support to the idea that the left hippocampal activation during correct answering in our study might have reflected a cued recollection experience. Similarly to Mendelsohn et al. (2010), we could argue that the statements presented during the fMRI-scanning acted as verbal cues for mentally recasting and recollecting the material presented in the film. On the other hand, several authors that looked at the hippocampus and processing of novel information (Tulving et al., 1994), found that the left hippocampus was activated during conditions that contained novel information (verbal or pictorial) (Poppenk et al., 2008; Hashimoto et al., 2012). These results are interesting in the light of our behavioral findings; as mentioned above, when participants answered a statement correctly, this was much more frequently the consequence of a correct rejection than of a hit. According to this line of thought, the left hippocampal activation during correct answers may alternatively (or additionally) reflect a correct rejection of novel, unstudied material (Düzel et al., 2003). One could speculate that the activation of the hippocampus may have indicated the detection/encoding of novel material (Düzel et al., 2003; Friedman et al., 2011). Alternatively, the observed activation might have signified a recollection rejection strategy, which is a plausible strategy in this population with intact working memory capacity (Koriat et al., 2008; Leding, 2012).

In our study, we also found activation in the lingual gyrus, an area that was described to be more active for correct than for incorrect (lag) judgments (Greve et al., 2010). The lingual gyrus was described as being part of the default-mode network and has been implicated in the generation of visual mental images, visual details of actual past events and “image content that is accessed via verbal materials” (Greve et al., 2010, p. 7103). Leshikar et al. (2012) reported task-selective memory effects for visual imagery (a monotonic increase in activity according to vividness) in the left precuneus as well as left occipital and right lingual gyri. In a functional imaging study by Gilboa et al. (2004) context-rich memories were associated with increased activity in right precuneus and bilateral lingual gyrus independently of remoteness. Addis et al. (2009) identified activations of lingual gyrus (and other posterior visual areas) during recall of actual past events as well as during construction (imagination) of past or future episodes; however, the activation was higher during the first condition in comparison to the last two conditions. The higher activation of the lingual gyrus during the recall (recollection) of actual past events was interpreted as being in line with the sensory reactivation or reinstatement hypothesis (Nyberg et al., 2000; Wheeler et al., 2000; Schacter and Loftus, 2013).

Another interesting result yielded by our study concerns the bilateral activation of the precuneus in combination with the hippocampus. This result is related to the finding of hippocampal connectivity to the left precuneus in a recollection network (in contrast to familiarity) (Dörfel et al., 2009). Increased activity of precuneus was demonstrated for recollected items relative to misses, correct rejections, and strong familiarity (Kafkas and Montaldi, 2012). In a recent analysis, Kim (2013) showed that default-mode network areas, including the left precuneus, exhibited greater old/new (hit more than correct rejection) effects during a source-retrieval testing paradigm than during an item-retrieval task. This was interpreted as reflecting an enhanced ecphoric processing during the first testing paradigm (Kim, 2013). Addis et al. (2009) demonstrated via fMRI precuneus activations during both recollection of actual past events and construction of past or future events; however the construction-related tasks were accompanied by “greater percent signal change” in precuneus in comparison to the recollection-task. A PET study that investigated the neural correlates of true autobiographical memories and fictitious autobiographical memories found that fictitious autobiographical memories were associated with higher activations in the (left) precuneus than true autobiographical memories (Markowitsch et al., 2000). Cavanna and Trimble (2006) advanced the idea that there may be a functional dissociation within the precuneus; in particular, they proposed that the posterior precuneus may be associated with successful retrieval attempts, while the more anterior portion may be engaged in the retrieval mode via mental imaging. Memory-related imagery was in fact associated with significant activations of the anterior precuneus bilaterally in a seminal study, using positron emission tomography, conducted by Fletcher et al. (1995). In a recent fMRI study, Huijbers et al. (2011) however found the ventral precuneus to be associated with successful retrieval, but with unsuccessful imagery performance (including auditory imagery performance). The authors subsequently speculated that the activation of ventral precuneus during unsuccessful imagery may have reflected the processes of generation and comparison of alternative mental representations. The recruitment of precuneus areas during the generation and mental inspection and matching of alternative representations (Markowitsch et al., 2000; Kühnel et al., 2008; Hirshhorn et al., 2012) may offer an explanatory avenue for observed activations of precuneus regions not only during the RP (correct versus incorrect answering), but also during the MP (low confidence judgments versus high confidence judgments) of our study. In the latter case, it could be argued that the activation of the precuneus reflected the use of conscious visual imagery strategies, in an attempt to facilitate retrieval (Fletcher et al., 1996; Cavanna, 2007; Koriat et al., 2008; Desseilles et al., 2011).

The left MTG was found to be connected with memory recollection (Fink et al., 1996; Kroll et al., 1997; Markowitsch et al., 2000; Botzung et al., 2008; LePort et al., 2012) and essentially in the comparison between misses and correct rejections (Takahashi et al., 2008), which is reflected in the behavioral data insofar as the contrast correct versus incorrect responses was represented by correct rejections versus misses. This is supported by the importance of the medial temporal lobe in consolidation and retrieval of recently learned items (Cabeza and Nyberg, 2000; Sybirska et al., 2000; Frankland and Bontempi, 2005; Botzung et al., 2008). The observed activation of the insular cortex may reflect the posited contribution of the insula (especially of its anterodorsal part) to attentional processes, speech production, and memory recall (Manes et al., 1999; Nieuwenhuys, 2012).

Based on the current results we conjecture that a network of memory-related regions, including the hippocampus, the precuneus, areas within the posterior visual cortices (lingual gyrus), and other areas within the MTG, subserves correct (in contrast to incorrect) answering in the RP, at least at delays of around 2 h.

The contrast incorrect answering versus correct answering did not yield any significant results in our study. It can therefore be speculated that incorrect answering is not supported by a distinct neural net, but it is widely sustained by the same reconstructive approaches that support the correct answering. Incidentally, a study that investigated the neural correlates of false in comparison to true memories and vice versa via both functional connectivity analysis and direct contrasts, failed to identify any brain region displaying more activity during false versus true remembering (recollection) in direct contrasts; however, differences among the two conditions were suggested by connectivity analysis (Dennis et al., 2012).

### Monitoring process (high confidence versus low confidence)

The factor MP (monitoring; high confidence versus low confidence) revealed no significant effect in the behavioral rating during the scanning procedure; hence there are no statistically significant differences between responses given with high confidence and responses given with low confidence. Imaging data draw a different picture: When contrasting high subjective confidence (defined here as absolute sureness) against low confidence, activation was particularly located in the fusiform gyrus (bilaterally), the left lingual gyrus, and the left parahippocampal area (Botzung et al., 2010). One might therefore argue that absolute sureness about a given answer was to a prevailing degree elicited by the accessibility (fluency) of rich visual details during the mental recasting or recollection of episodic-like material (Wheeler et al., 2000; Greenberg and Rubin, 2003; Greenberg, 2004; Kensinger and Schacter, 2006; Daselaar et al., 2008; Addis et al., 2009; Chua et al., 2012). Greenberg (2004) remarked that “when people retrieve visual images” (p. 367) they tend to be more confident in the veracity of their memories. The activation of the fusiform gyrus has been related to the visual processing of faces (but also objects and words) and that of the parahippocampal region to place perception (Kanwisher, 2010; Hofstetter et al., 2012), processing of scenes and landmarks (Piefke et al., 2003, 2005; Sharot et al., 2007), fine grained spatial judgments (Hirshhorn et al., 2012), and reinstating of visual context to facilitate successful retrieval (Hayes et al., 2007). Furthermore, it is worth mentioning that parahippocampus and ultimately hippocampus receive a diverse gamut of synthesized sensory-specific in addition to multimodal cortical information (Nieuwenhuys et al., 2008). Faces, objects, and places were of course frequently present in the movie and consequently a common subject of the questionnaire. Incidentally, confidence effects were related to parahippocampus in a recent study by Hayes et al. (2011). In this study, in the source, but not item memory task the high versus low confidence contrast induced activation in the right hippocampus, extending to parahippocampal cortex. Furthermore left parahippocampal activations were elicited for high confidence judgments versus low confidence judgments in an fMRI study that used a modified version of the Deese-Roediger-McDermott (DRM) (Roediger III and McDermott, 1995) paradigm (Moritz et al., 2006).

In our study, the reverse contrast (low confidence versus high confidence judgments) revealed activations within the same regions and in addition a broad pattern of diffuse frontal, temporal, parietal, occipital, and limbic activation. Interestingly, other recent fMRI-studies also reported brain regions common to high and low confidence recognition, indicating the contribution of these regions to both high and low confidence recognition activity (Kim and Cabeza, 2009; Hayes et al., 2011). The recruitment of the same brain regions by both high and low confidence ratings may be due to several factors, such as a division of labor (a functional dissociation or heterogeneity) among various parts of these regions (Hofstetter et al., 2012). The widespread activation of brain regions accompanying low confidence ratings may be interpreted as reflecting processes of increased and effortful allocation of attention, executive control, searching, mental inspection, matching, and self monitoring resources.

Significant neural activity was found in our analysis in the posterior and middle cingulate cortex for low confidence judgments. This finding partly overlaps with the results of Hayes et al. (2011). In their study the posterior cingulate cortex was activated during high confidence judgments, while the middle portion of the cingulate cortex was activated during low confidence judgments. Differentiated activations within cingulate regions, concerning comparisons of high versus low confidence judgments, were also described by Chua et al. (2006). The modulation of posterior cingulate cortex by confidence judgments could be related to its converging position within the default-mode network, which enables it to integrate mnemonic information with aspects derived from internally oriented mentation, such as self-referential (e.g., self monitoring), emotional, and social information (Kim, 2013; Chua, 2012). The middle cingulate cortex has been assigned functions in response selection or decision, such deliberating in a volatile environment (Chiu et al., 2008; Frith and Frith, 2008). Huijbers et al. (2011) advanced the idea that the midcingulate cortex, supramarginal gyrus, and precuneus areas contribute to the mental inspection of competing/alternative mental representations, which may explain the activations of these areas in the low confidence condition versus high confidence condition of our study.

Henson et al. (1999) performed an event-based functional MRI study, during which they asked volunteers to make one of three judgments to each presented word during recognition. Subjects had to judge whether they recollected seeing it during study (R judgments), whether they experienced a feeling of familiarity in the absence of recollection (K judgments), or whether they did not remember seeing it during study (N judgments). The R and N judgments can be assumed to be analogical to the high and low confidence rating in this study. Henson et al. (1999) found increases for N judgments (in contrast to R judgments) in the middle and superior frontal gyrus, insula, amygdala, precuneus, inferior parietal gyrus, and MTG. Except for the amygdala exactly the same areas were found to be activated in the current study when participants rated statements with low confidence (as opposed to high confidence). It is actually easy to imagine that subjects rated a statement with low confidence when they did not remember the accordant item. With respect to amygdala some studies proposed that increased activation may be associated with increased vividness, intensity of emotional judgments and self-relevance of memory, and memory confidence (for a review see Markowitsch and Staniloiu, 2011a). In our study we did not detect amygdala activation in association with either contrast during confidence ratings. The lack of amygdala activation (Vuilleumier et al., 2004) may reflect the process of habituation, such as in blocked fMRI designs (Greenberg et al., 2005; Daselaar et al., 2008). Another interpretation could be that the modulation of amygdala by confidence judgments may vary as a function of valence (Botzung et al., 2010).

Our data suggest that confidence ratings in general modulate temporal and occipital areas, which participate to gathering and integrating information (evidence) from various perceptual/sensory areas (Kim and Cabeza, 2009; Huijbers et al., 2010). These areas may show different modulations in relationship to valence, arousal, and novelty of mnemonic information and task characteristics. Furthermore, the relation between confidence and emotional intensity may vary as a matter of valence (Botzung et al., 2010). For positively valenced visual material (movie shots) associated with high confidence, Botzung et al. (2010) found increased activity was found in the medial temporal lobe as well as in the insula.

On the other side, specific frontal regions [inferior frontal gyrus (IFG), superior and middle frontal gyrus, paracentral lobule, precentral gyrus] were associated with low confidence in comparison to high confidence judgments, in our study, possibly reflecting executive control processes (Kim and Cabeza, 2009). The activation of the left IFG was connected to the deployment of controlled retrieval operations (Oztekin et al., 2009), especially when remembering is more difficult (Kim and Cabeza, 2009). Incidentally, the left IFG was detected to be activated in response to cues that elicit a strong need for selection among competing representations (Zhang et al., 2004; Moss et al., 2005). Furthermore higher activity at IFG was found to be correlated with higher risk aversion (Christopoulos et al., 2009) and the left IFG was attributed an essential role for suppressing prepotent, but inappropriate answers (Swick et al., 2008). One could speculate that the presence of competing mental representations may be conducive to a higher risk aversion, which may get translated into avoiding the 100% confidence option. When choosing the 50% confidence option subjects made no strong commitment and there was no risk.

Further activation during low confidence judgments was found in our study in the superior frontal gyrus being related to Brodmann area (BA) 10, which is assumed to play a role in strategic processes involved in memory retrieval and higher cognitive function (Burgess et al., 2007). This result again emphasizes the role of executive control processes in decision-making under uncertainty. We assume that when subjects are uncertain about a memory engram they invest more effort in retrieval of mnemonic information, which requires higher cognitive functions and is correlated with a rather ambivalent activation pattern compared to high confidence.

Some authors identified a laterality-confidence effect within frontal cortex. Right ventrolateral prefrontal regions were more active during low versus high confidence for both item and source memory tasks, supporting a role of this area in the processing of weak memories (Hayes et al., 2011). In the same study, several left prefrontal cortex regions showed greater activity for source than for item memory, independent of confidence.

When using a statistical height threshold of *p* < 0.05, FWE corrected for multiple comparisons only the left precuneus was activated in our study during low confidence versus high confidence ratings, a finding that mirrors results of other authors (Kim and Cabeza, 2009). Previous fMRI-studies have demonstrated that the precuneus (and posterior cingulate areas) show greater activity during recollection-based judgments (Henson et al., 1999; Wagner et al., 2005). In the study of Botzung et al. (2010) emotional intensity modulated the activity of the precuneus; furthermore, Sharot et al. (2007) found that precuneus activation correlated with the personal relevance during memory retrieval. In an elegant fMRI study that looked at the spatio-temporal dynamics of episodic-autobiographical memory, Daselaar et al. (2008) found that precuneus activation occurred at the elaboration phase of retrieval, after a specific memory had already been selected. Some authors connected the precuneus to gathering and integrating sensory details during watching movie sequences from silent films (Hasson et al., 2008). The precuneus was described as being part of the retrieval success network and was portrayed as being a sensory evidence accumulator (Huijbers et al., 2010).

In contrast to the view that holds the precuneus as being part of the retrieval success network, a recent study provided evidence that precuneus might be more implicated in retrieval confidence (decision-related retrieval processes) than successful episodic retrieval (Huijbers et al., 2010). The role of the precuneus in decision-making under uncertainty had also been suggested by Paulus et al. (2001), who asserted that both “STG and precuneus have been associated with sub processes that are consistent with the maintenance of strategies in the presence of uncertainty” (p. 97).

In conclusion, many more and differential brain areas were activated in our study when contrasting low against high confidence, a difference we attribute to greater monitoring demands and allocation of cognitive resources when confidence judgments are made under uncertainty.

### Control process (volunteering versus withholding)

The factor CP (control; volunteering versus withholding) induced a significant effect in the behavioral rating during the scanning procedure: subjects rather volunteered an answer instead of withholding it, which is understandable due to the fact that each correctly volunteered answer was rewarded with a bonus, while volunteering an incorrect response led to a loss of one bonus point (moderate incentive). When no answer was provided (volunteered), no penalty or reward was instituted (no bonus points were gained or lost). The imaging data revealed differential activation for volunteering and withholding, suggesting that the two forms of behavior engage at least partially distinct sets of cognitive processes. If an answer was volunteered, particularly temporal and frontal as well as middle and posterior cingulate areas and the precuneus revealed activation. These data suggest that activation for volunteering is very similar to the neural correlates of monitoring (in contrast to retrieval). The findings are not surprising as they point to a relationship between memory confidence and answer volunteering (Koriat et al., 2008). Confidence judgments are assumed to be based on the strengths of the underlying memory trace. Also vivid remembering leads to making a high confidence decision. Volunteering an answer however might be even more influenced by perceived vividness of remembering due to the accompanying situational demands and payoffs (Belli et al., 1994; Yonelinas, 1994; Busey et al., 2000; Bradfield et al., 2002; Shaw and Zerr, 2003).

In contrast, when the answer was withheld, activations in parietal and temporal regions and also in the left caudate nucleus were identified. This may come as a surprise, because due to the task demands which were assumed to be connected with control processes like executive functioning, rather frontal and prefrontal brain activity would have been expected to reveal activation (Hedden and Gabrieli, 2010). The relevant brain areas for response inhibition which we anticipated to be related to withholding an answer include the ventrolateral PFC, mainly in the right hemisphere often in conjunction with a more extensive fronto-striato-parietal network (Garavan et al., 1999; Konishi et al., 1999; Aron et al., 2004; Walther et al., 2010; Ghahremani et al., 2012).

In our study we found during answer withholding a substantial activation in areas that play a differential role in memory retrieval (Takahashi et al., 2008; Hoscheidt et al., 2010). This might suggest that subjects were more concentrated on the reconstruction or re-retrieval of the appropriate memory instead of response selection/inhibition (Robbins, 2007). This may have been caused by the design specification, because subjects had only very short time to answer. There might have been an overlap with the retrieval process especially when someone was not sure about the memory. We subsequently observed bilateral hippocampus activation, which speaks in favor of a great allocation of resources toward mentally reconstructing and generating internal memory details or contextual details that can be used as a retrieval cue (Koriat et al., 2008).

Caudate nuclei activity was found to be modulated by performance-feedback, including monetary rewards, with greater activation being observed during high confidence recollective experiences (Kim, 2013). However, in our study the identification of increased activation in the left caudate during withholding of answering might be congruent with new data showing the involvement of the left caudate in the inhibition of unwanted responses (Badgaiyan and Wack, 2011).

The meaning of activation of the left Heschl gyrus during withholding answers is unclear. It may reflect cognitive strategies or attempts at mental reconstruction (Zarnhofer et al., 2012). Imagining speech in third person was among other associated with left sided activation in STG and left postcentral gyrus (Shergill et al., 2001). During a visual imagery task Stokes et al. (2009) found activation of the superior temporal sulcus/Heschl’s gyrus, but they speculated that it may have reflected the existence of auditory cues. Huijbers et al. (2011) however found overlapping activations in auditory cortex/ STG for auditory perception, retrieval, and imagery. The left postcentral gyrus activity was reported in one study in association with both novelty detection activity and encoding failure activity (Kim et al., 2010).

### Monitoring process versus retrieval process

The contrast between monitoring and retrieval resulted in temporal, occipital, parietal, and frontal brain activation, and moreover bilateral activation of the precuneus and differential left cingulate cortex areas. While during the RP subjects rated each statement as correct or incorrect, the MP was defined as a confidence judgment. During the confidence judgment, subjects monitored their recognition decision, and made an explicit subjective judgment about their previous memory performance. It is common sense to make the assumption that both processes (RP and MP) are related, although experience shows that there are instances where subjective confidence and objective correctness of memory answers divert (Simons et al., 2010). As Chua et al. (2009) expounded, confidence judgments are considered to be based on the strength and/or quality of the underlying memory trace, ease of retrieval, and also on study specific heuristics and test conditions, and ultimately on participants’ general mnemonic abilities (Belli et al., 1994; Yonelinas, 1994; Busey et al., 2000; Bradfield et al., 2002; Shaw and Zerr, 2003). The functional imaging study conducted by Moritz et al. (2006) reported an increase in confidence at recognition associated with bilateral activation in the anterior and posterior cingulate cortex along with medial temporal regions. In comparison to recognition judgments, confidence judgments induced higher activations in various regions (such as superior frontal, dorsomedial frontal, orbitofrontal, and lateral parietal cortices), including areas involved in self-referential processing and internal mentation (such as medial prefrontal cortex) in an fMRI study conducted by Chua et al. (2006). Our own results reveal that, when contrasting confidence judgments to retrieval, activations are detected in areas involved in generation and inspection of mental imagery (e.g., precuneus, middle frontal gyrus, middle cingulate gyrus, supramarginal gyrus; Huijbers et al., 2011; Hirshhorn et al., 2012), monitoring and detecting conflict [e.g., anterior cingulate cortex (Acc)], post-retrieval monitoring and verification (prefrontal areas), decision and response selection under uncertainty (e.g., middle cingulate cortex, Frith and Frith, 2008), cognitive dissonance (e.g., Acc; van Veen et al., 2009), self appraisal (e.g., medial prefrontal cortex areas, Ries et al., 2012), and motivation and emotional processing (e.g., Acc). Activation of the Acc may support the relationship between monitoring processes and executive functions, given that the dorsal part of the Acc is connected with the prefrontal cortex, which plays a crucial role in executive functioning. Incidentally, Fan et al. (2005) described a kind of executive control network that showed activation of the anterior cingulate along with other brain areas.

The reverse contrast (RP versus CP) did not yield any statistically significant difference in our study. One may conclude from this that, even though monitoring and retrieval processes are strongly connected with each other, the former is characterized by a higher demand for cognitive performance and an additional allocation of resources toward internal reflection, inspection, verification, and comparison of alternatives and self-referential processing (Chua, 2012).

### Control process versus retrieval process

According to our results, the CP (in contrast to retrieval) revealed (amongst others) the same neural activation as monitoring (in contrast to retrieval), namely the left MTG and the middle frontal gyrus. The left MTG was found to be activated during episodic-autobiographical memory retrieval (Markowitsch et al., 2000), but also during a variety of semantic tasks (for a review, see Svoboda et al., 2006). The MTG is indeed portrayed as an information convergence hub, as it integrates auditory and visual information (Visser et al., 2012). The left MTG is considered to be a crucial node of the conceptual network and has been attributed roles in word-picture matching, mapping concepts to words, and semantic task decision. Its recruitment during retrieval of old episodic memories was conjectured to support the idea of multimodal representation of episodic memory (Fink et al., 1996), on one hand and the more complex and effortful process of ecphorizing, on the other hand (Markowitsch et al., 2000). As mentioned above the left MTG was found to be activated during correct answering versus incorrect responding during the RP (Takahashi et al., 2008), which is in line with its involvement in the monitoring and CP, however the outcome of both processes is based on recognition decision (Chua et al., 2009).

### Memory accuracy

The factor MAC (high MAC versus low MAC) induced differential brain activation for both contrasts. High MAC was here defined as withholding an incorrect answer in contrast to volunteering an incorrect answer. The present neural activation related to MAC (in contrast to inaccuracy) again confirms our previous assumption of a network of memory-related areas including the hippocampus, the precuneus, and the MTG being related to correct (in contrast to incorrect) memory retrieval hence these areas showed activation. The important role of the medial temporal lobe (including the parahippocampal gyrus) in memory performance and, particularly in non-verbal memory, has been known since the mid-1950s (Scoville and Milner, 2000; Frankland and Bontempi, 2005).

The reverse contrast, low MAC (in contrast to high MAC), was defined as volunteering incorrect answers. Interestingly, only the left superior frontal gyrus and the left insula demonstrated neural activation during low MAC in comparison to the high MAC condition. Mohr and colleagues investigated the role of the insula, concluding that this region was consistently associated with risky behavior (Weller et al., 2009; Mohr et al., 2010). Moreover the insula was found to be predominantly active in the presence of potential losses (Mohr et al., 2010). Insular activations were also reported in relationships to unexpected outcomes and errors (Klein et al., 2013). Low accuracy was defined in our study as volunteering an incorrect answer. If an answer rated with a confidence less that 100 percent were ventured, subjects would experience a homeostatic and visceral change in the face of potential losses, which would lead to insula activations. This would be in accordance with our experimental design hence participants knew that incorrect decisions would result in a loss of bonus and moreover therewith tended to a risky decision. Early studies had shown that people are loss aversive; however newer data pointed to a reduction (or even reversal of loss aversion) if people anticipate gains and losses and the anticipated loss is small (Harinck et al., 2007). The activation of the left superior frontal gyrus was detected in an imaging study when contrasting conceptual false to conceptual true information (Garoff-Eaton et al., 2007). In another study, activations of superior frontal gyri were reported to signify loss aversion when making decisions under risk (Tom et al., 2007; Xu et al., 2009). In our study the left superior frontal gyrus was also modulated when contrasting low confidence with high confidence ratings. It is therefore possible that uncertainty and inaccuracy belong to related processes.

## Conclusion

In a 7 Tesla fMRI study that adopted the paradigm developed by Koriat and Goldsmith (1996a,b) and used complex, emotional, naturalistic, and culturally appropriate material at encoding (the movie “The New Cat”), we have provided evidence for common and unique neural correlates underlying the processes of memory retrieval, monitoring and control, and MAC performance in a group of healthy young adults. The participants were well matched for educational background and neuropsychological performance and equally distributed with respect to sex. The administration of the memory queries about the movie, which took place after a period of interference of about 2 h, tried to approximate the real-life situations related to eyewitness testimony. The 2-h period between seeing the short movie and being tested in the scanner was completely filled by neuropsychological testing and pre-scanning procedure; therefore the participants had no possibility to recapitulate details or to talk about the film. Furthermore, as it was mentioned above the participants were not informed that their memory of the movie would be probed later – a condition that again tried to approximate real-life eyewitness testimony circumstances.

As expected, the correct answering versus incorrect responding in the RP was accompanied by increased activation in hippocampus (Habib and Nyberg, 2008). The material used for incidental encoding involved complex multisensory information and it is known that information coming from all sensory modalities is transmitted to the hippocampal formation. In our study, we found a left lateralization of hippocampal engagement; this finding is relevant given data supporting the involvement of the left hippocampus during the retrieval of strict episodic memories in response to a specific cue.

Strict episodic memories are characterized by increased vividness, perceptual details, emotional engagement, self-relevance, and autonoetic consciousness (Markowitsch and Staniloiu, 2011b, 2012). The latter entails mental time traveling and reliving of the contextual details from the time of the encoding.

An alternative explanation is that the left hippocampal activation during correct answers (in comparison to incorrect answers) may have indicated a correct rejection of novel, unstudied material (either the detection/encoding of new material or a recollection rejection strategy). In our study, subjects gave more correct than incorrect answers, however, this was in general a consequence of correct rejection (instead of a hit) (McDonough and Gallo, 2012). The left hippocampus was also modulated in our study during the contrast high MAC versus low MAC.

One can assume from the results related to monitoring processes that temporal areas are involved in confidence ratings in general, whereas particular frontal regions are associated with low confidence judgments. The activation of the parahippocampal gyrus during confidence memory ratings is congruent with data showing that, similarly to hippocampus, parahippocampus receives a large gamut of sensory-specific and multimodal cortical information. The parahippocampus has been involved in retrieving non-verbal material and other authors evidenced its activation in relation to confidence memory judgments (Hayes et al., 2011). The finding of a significant positive association between left precuneus activation and the contrast low confidence versus high confidence judgments is consistent with other reports pointing to the recruitment of precuneus areas during post-retrieval monitoring processes (Huijbers et al., 2010).

In our study, answer volunteering seemed to be subject to increased monitoring processes, in contrast to answer withholding. These monitoring processes (suggested by activations in prefrontal, cingulate, and parietal cortices) may be modulated by the amount of potential gain relative to loss.

The increased bilateral hippocampal activation associated with withholding answers may reflect the posited role of hippocampus in disambiguation, mental construction, prospection, and future-minded choices (Ross et al., 2009; Peters and Büchel, 2010). As withholding of information may represent low memory confidence and/or reduced memory vividness, there may be a need for inter-hemispheric engagement of hippocampal formation, in order to generate and bind together pieces of information that are sensorially varied and complex (Botzung et al., 2010).

The caudate nuclei, which are part of the reward system, are assumed to support satisfaction linked to target detection (especially in relationship to hits) (Kim, 2013), monetary gain, and acquisition of good reputation (Izuma et al., 2008). In our study, we only found a left caudate activation during withholding of answering, but we interpreted this differently, namely by relating it to the involvement of the left caudate in the inhibition of unwanted responses (Badgaiyan and Wack, 2011).

The present study has a number of limitations; therefore its results cannot be generalized to the complex eyewitness situations. The short emotional movie only established a bridge between old laboratory memory testing and real-life situations, by inducing a controlled experience, with elements of real-life events (Mendelsohn et al., 2010). In real life, a much more variable mismatch between encoding situations and conditions at the time of memory testing might exist. The delay between learning and testing was about 2 h in our study. In eyewitness testimony situations, variable delays might be encountered. The observed neural correlates associated with some of the processes described above might not hold true at longer testing delays (Schacter and Loftus, 2013). A higher degree of homogeneity characterized the population investigated in our study, which obviously is not the case in eyewitness cases. Furthermore, we used a modest incentive during the CP of our study, whereas higher incentives may be involved in eyewitness testimony settings.

Despite its limitations, the present study demonstrates that dissociation between the retrieval, monitoring, and control processes described by the paradigm of Koriat and Goldsmith is realizable at the neurobiological level. We found a network of memory-related regions, including the hippocampus, the precuneus, and the MTG playing a crucial role in correct memory retrieval (correct answering during the RP) and MAC. Moreover, we show evidence for the fact that volunteering may be connected with monitoring processes hence both seem to be based on the strengths of the accordant memory trace. Our results reveal a strong relationship between monitoring and retrieval processes, whereas monitoring is defined by a higher demand for cognitive performance.

Beyond that, monitoring and control processes have in common that their outcome is based on a recognition decision which reflects their strong connection to memory retrieval – this relation seems to be mediated by activation of the medial temporal gyrus.

## Conflict of Interest Statement

The authors declare that the research was conducted in the absence of any commercial or financial relationships that could be construed as a potential conflict of interest.

## Footnotes

^1^http://www.neurobs.com

^2^http://www.fil.ion.ucl.ac.uk/spm

## Appendix

### Examples of sentences used for testing remembrance

#### Correct statements

1. The car of the woman is white.
2. He reads a Hebrew newspaper.
3. The couple buys for themselves a dog.
4. He calls the emergency service, when the cat jumps from the window.
5. She cries after the accident.
6. He gives vitamins to the cat.
7. He gives dog food to the cat.
8. He cleans the window with a cloth.
9. He was already once bitten by a dog.
10. He puts a dog bone on the grave.
11. Pictures hang above the TV set.
12. He reads the newspaper in the toilet.
13. She asserts that she drove slowly.
14. The curtains of the main character are checkered.
15. The cat is brown-beige.
16. He sleeps with the cat in bed.
17. In the kitchen there is a white water kettle.
18. The couple speaks about the professional carrier of the woman.
19. In the kitchen there is a pan on the oven.
20. In the kitchen there is a fan on the ceiling.

#### Incorrect statements

21. He has a roommate.

22. She wears a white top.

23. At the grave he kisses the woman.

24. She wears a skirt.

25. He reads a book.

26. She says that she drove too quickly.

27. He plays a videogame.

28. The bowl of the cat is red.

29. The couple sleeps with each other without the dog in bed.

30. The new dog’s name is Bobby.

31. His friends eat Pizza in his apartment.

32. When he drives he wears no glasses.

33. The balloons from the room have different forms.

34. He offers the cat spaghetti at breakfast.

35. She puts a bunch of flowers on the grave.

36. The vacuum cleaner is blue.

37. In the kitchen hangs a chandelier.

38. He yells at the woman, when he sees her.

39. There are socks under the bed.

40. He makes phone calls in the toilet.
